# A web-based cross-sectional study assessing the impact of COVID-19 on the mental health of radiology staff in Saudi Arabia

**DOI:** 10.1371/journal.pone.0265873

**Published:** 2022-04-04

**Authors:** Rawan Abdeen

**Affiliations:** Diagnostic Radiology Department, King Abdulaziz University, Jeddah, Saudi Arabia; The University of Jordan, JORDAN

## Abstract

The 2019 novel coronavirus pandemic has not only created massive public health issues, it has also produced excessive psychological disorders in healthcare professionals, including radiology staff. The aim of this study is to assess the risk perception and mental health of radiology staff in Saudi Arabia during the COVID-19 pandemic. The researcher asked radiology staff to complete an online Google Forms questionnaire, between June 10, 2020 and June 17, 2020, which contained demographic data and self-designed questions related to anxiety, insomnia, depressive symptoms, and mental health services during the pandemic. A total of 168 radiology staff participated in the study. The results indicated that 53.05% and 57.14% of the participants were experiencing mild to severe symptoms of anxiety and depression, respectively. Moreover, 47.02% of the participants were experiencing insomnia symptoms. Among all the participants, only 16.61% had received psychological materials from their hospital during the pandemic, while 22.02% had accessed online psychological assistance techniques. The health of roughly one-third (30.95%) of the participants was worse than it had been before the pandemic. COVID-19 is a source of mental health disorders for healthcare professionals, particularly radiology staff. The findings of this study indicate that more than 70% of radiology staff in Saudi Arabia are concerned about insufficient protective measures and the risk of infection. In addition, a large percentage of them have experienced mental health disorders, such as anxiety, insomnia, and depression. Regular mental healthcare services are required to decrease the negative impact of the pandemic and enhance the overall mental health of the radiology staff.

## Introduction

We are in the midst of a global health crisis. From the time it was discovered in December 2019 in Wuhan, China, the novel coronavirus known as Severe Acute Respiratory Syndrome Coronavirus 2 (SARS-CoV-2), which causes the Coronavirus Disease 2019 (COVID-19), has affected millions of people throughout China and around the world [1, 2]. In January 2020, the World Health Organization (WHO) declared the disease caused by the virus to be a public health emergency and, by March 2020, a pandemic [3]. There has been a noticeable increase in mortality and morbidity in the last few months, due to the pandemic. As of 14 July 2020, Saudi Arabia had recorded about 235,111 total confirmed cases, 2,243 deaths, and 169,842 recoveries [4]. As this infectious disease has continued to spread around the world, healthcare professionals have been largely responsible for screening and treating patients [5].

During the COVID-19 pandemic, a great number of patients with suspected or confirmed cases of COVID-19 have been directed to hospitals [6]. Therefore, the examination workload in radiology departments has sharply increased and radiology staff are facing massive pressures, including increased workloads, isolation, exhaustion, a high risk of becoming infected, and a lack of contact with their families (as the coronavirus is known to be transmitted through close contact and respiratory droplets) [6, 7]. Radiology staff, including radiologists, radiology technologists, radiology technicians, radiology nurses, and receptionists, are front-line healthcare workers who have a high potential of being exposed to COVID-19 [8].

This pandemic not only influences physical health, but mental health and wellbeing as well [9]. Over the last few months, mental health problems have increased among patients infected with COVID-19, as well as the general population, college students, and healthcare professionals [10, 11]. Mental health disorders can affect the concentration, understanding, and decision-making capabilities of radiology staff, which might hinder the fight against COVID-19 and have long-term effects on their overall health [7]. Several previous studies that examined the effect of the Ebola and SARS outbreaks on healthcare professionals found that outbreaks cause severe psychological disorders that influence workers’ overall wellbeing [12, 13]. Greatly increased workloads, physical fatigue, and insufficient supplies of protective materials can impact the physical and mental health of healthcare professionals [14]. Previous research during the SARS pandemic found that healthcare workers were at a high risk of developing depression, stress, and anxiety [15]. Some researchers have similar thoughts regarding the impact of the COVID-19 pandemic, which can give rise to disorders such as insomnia, stress, depression, and anxiety [16–18]. Several studies in recent months have evaluated the impact of this disease among healthcare professionals [16–18]. Zhang et al. found that 36.1% of healthcare workers have displayed symptoms of insomnia during the COVID-19 outbreak [18]. Moreover, Liang et al. reported that many healthcare workers demonstrated significant symptoms of clinical depression [19].

There have been inadequate psychiatric screening and counselling services to treat insomnia, depression, and anxiety in radiology staff during this pandemic [10]. Most authorities focus mainly on the biological and physical effects of the pandemic on the population, but ignore the needs in the mental health domain [5]. Therefore, it is important to protect the mental health of healthcare professionals to control the pandemic and maintain their long-term wellbeing.

During the novel COVID-19 pandemic, healthcare providers in Saudi Arabia have continued working and risking their lives out of their professional and ethical obligations. As a result, they have been under near-constant psychological distress. Recent studies explored the mental health of healthcare providers in Saudi Arabia during the COVID-19 pandemic. AlAteeq et al. found that more than half of healthcare providers had generalized anxiety and depression (51.4% and 55.2% respectively) [20]. Al Mutair et al. found that 43.5% of healthcare providers in Saudi Arabia suffer from anxiety [21]. On the other hand, it was demonstrated that non-Saudi healthcare providers in Saudi Arabia experienced high levels of depression and anxiety compared to their Saudi peers [22]. This could be due to the fact that non-Saudi healthcare providers live alone and far away from their families. Moreover, cultural norms and differences in living conditions between Saudi and non-Saudi healthcare providers could also affect mental health status [22].

A novel systemic review and meta-analysis of the prevalence of mental disorders among healthcare professionals highlighted a high prevalence of depression, anxiety, and insomnia during the COVID-19 pandemic among healthcare professionals [14]. A recent study found that about 55.2% and 51.4% of Saudi healthcare providers had symptoms of depression and anxiety during the pandemic. Similarly, depression and anxiety were experienced by 50.5% and 46.7%, respectively, of ophthalmologists in Saudi Arabia. AlAteeq et al. found that the prevalence of severe anxiety among healthcare providers during COVID-19 was twice that of the prevalence among emergency healthcare workers before the pandemic [20].

However, an extensive literature search revealed that no substantial survey has been conducted to evaluate the impact of the COVID-19 pandemic on the mental health of radiology staff in Saudi Arabia. Thus, it is necessary to evaluate and understand the mental health ramifications of COVID-19 and identify possible actions to cope with the pandemic and effectively manage public health. Supporting the mental health of radiology staff is a significant aspect of the public health response. Therefore, this research is aimed to assess the impact of the COVID-19 pandemic on the mental health, particularly in relation to anxiety, insomnia, and depression, of radiology staff and offer possible strategies to avoid any future stresses that may occur as a consequence.

## Materials and methods

This study is approved by the Research and Ethics Committee of Applied Medical Science, King Abdulaziz University. No. FAMS-EC2021-16.

A cross-sectional, web-based survey that was pre-designed for radiology staff was conducted in Saudi Arabia from June 10, 2020 to June 17, 2020. The participants were selected from twelve regions of Saudi Arabia, including Mecca, Madina, Riyadh, Eastren, Qassim, Tabuk, Aseer, Hail, Jazan, Najran, Al-Baha, and Al-Jouf. Both male and female radiology staff (doctors, technologists, technicians, interns, nurses, and radiology receptionists), who were at least 20 years old, working in a radiology department during the pandemic, living in Saudi Arabia, and speaking either Arabic or English, were included in the study. Respondents in the target groups were sampled using the convenience sampling technique. The questionnaires were build using Google Document Forms in two languages (English and Arabic) and hosted automatically through a special uniform resource locator (URL). The questionnaire was pilot-tested on 11 participants and a slight modification was made to the wording to guarantee the clarity of the questionnaire. The URL of the questionnaire was sent to the participants via email, social media, and other online platforms. The researcher used Google Document Forms to avoid spreading COVID-19 through physical contact or airborne droplets. The questionnaire was anonymous (did not ask for names or email addresses) to ensure the confidentiality and reliability of the data being collected. Participation in the study was completely voluntary. All participants provided written informed consent electronically before participating. The informed consent page contained two options (Yes and No). Only the participants who selected “yes” could move on to the questionnaire. The system only counted responses with a 100% completion rate. The questionnaire required about five minutes to fill out completely.

The structured questionnaire packet contained questions on basic demographic data, including gender (male or female), age (in years), nationality (Saudi, non-Saudi), marital status (unmarried, married, divorced, widower), radiology career (doctor, technologist, technician, intern, nurse, radiology receptionist), specialty (X-ray, ultrasound, MRI (magnetic resonance imaging), CT (computed tomography), nuclear medicine, interventional radiology), work experience (years), and work shift (morning, evening, night, multi-shift). In addition, 11 self-designed questions assessed the participants’ risk perception of COVID-19.

The researcher also used three scales to evaluate the mental health status of the radiology staff. The seven-item Generalized Anxiety Disorder Scale (GAD-7), the seven-item Insomnia Severity Index (ISI), and the nine-item Patient Health Questionnaire (PHQ-9) were used to evaluate anxiety, insomnia, and depression, respectively. On all these scales, the higher the score, the more severe the symptoms.

The GAD-7 is a validated questionnaire that is increasingly used to assess anxiety among the general population as well as in primary care and research [23]. Each item is recorded on a four-point Likert scale from 0 to 3 (Johnson et al., 2019). The total score ranges from 0 to 21: minimal/ no anxiety is indicated by scores of 0 to 4, mild anxiety is indicated by scores of 5 to 9, moderate anxiety is indicated by scores of 10 to 14, and severe anxiety is indicated by scores of 15 to 21 [24].

The ISI is a valid, reliable seven-item self-evaluation psychometric tool intended to measure sleep difficulty. Each item is rated on a five-point Likert scale from 0 to 4 [25]. The total scores range from 0 to 28: absence of insomnia is indicated by scores of 0 to 7, sub-threshold insomnia is indicated by scores of 8 to 14, moderately severe insomnia is indicated by scores of 15 to 21, and severe insomnia is indicated by scores of 22 to 28 [25].

The PHQ-9 is a self-reported questionnaire used to evaluate the severity of depression in primary care and other medical settings [26, 27]. Each item is recorded on a four-point Likert scale from 0 to 21: minimal/no depression is indicated by scores of 0 to 4, mild depression is indicated by scores of 5 to 9, moderate depression is indicated by scores of 10 to 14, and severe depression is indicated by scores of 15 to 21 [26].

The final four questions were designed to assess mental healthcare services during the COVID-19 pandemic for healthcare professionals.

## Statistical analysis

The Kolmogorov-Smironov test was used to check the normality of the data. Descriptive analysis for the demographic data and mental health disorders (anxiety, insomnia, and depression) were documented as numbers and percentages. The means and standard deviations of anxiety, insomnia, and depression were also calculated. Moreover, anxiety, insomnia and depression scales were compared between individuals of varying genders, ages, and levels of work experience. For categories with two groups, an independent sample t-test was used. For categories with more than two groups (age and working experience), one-way ANOVA was used. Post-hoc analysis comparison of 95 confidence intervals of mean difference were conducted if ANOVA value was significant. Statistical Package for Social Sciences (SPSS) version 16.0 (IBM Corp., Armonk, NY, USA) was used for data analysis. The minimum sample size (n = 133) was calculated using G power software, version 3.1.9.4 (HHU, Germany).

## Result

A total of 168 questionnaires were completed and included in this study. Three participants refused to participate and were excluded from the study. In terms of gender, 52.98% of the participants were males and 47.02% were female. The age of the participants ranged between 20 and 69 years old, but a high percentage (56.55%) were between 26 and 35 years old. Most of the participants were technologists (63.09%) and about 44.64% specialized in X-ray technologies. Other demographic characteristics are shown in Table 1.

**Table 1:** Demographic characteristics of participants.

Variable	Category	Number (n)	Percentage (%)
**Gender**	Male	89	52.98
	Female	79	47.02
**Age**	20–25	46	27.38
	26–35	95	56.55
	36–45	27	16.07
**Nationality**	Saudi	158	94.05
	Non-Saudi	10	5.95
**Marital Status**	Single	91	54.17
	Married	74	44.05
	Divorced	3	1.79
**Radiology Career**	Doctor	14	8.34
	Technologist	106	63.09
	Technician	33	19.64
	Intern, Nurse, Receptionist	15	8.93
**Specialty**	Radiologist	13	7.74
	X-ray, Interventional	81	48.21
	MRI, CT, Ultrasound, NM	69	44.64
	Nurse, receptionist	5	2.98
**Working experience**	<5	106	63.09
	5–10	36	21.43
	>10	26	15.48
**Working shift**	Morning (8 am–3 pm)	85	50.60
	Evening (3 pm–9 pm)	1	0.60
	Night (9 pm–8 am)	3	1.79
	Multi-shift	79	47.02

MRI: Magnetic Resonance Image, CT: Computed Tomography, NM: Nuclear Medicine.

Table 2 reveals the participants’ responses to the questions related to the COVID-19 pandemic. The main concerns of the radiology staff were insufficient preventive measures to protect them against the virus (76.19%) and the current prevention and control strategy (71.43%). Moreover, 91.07% of the participants thought that working in the radiology field had increased their level of caution with regard to dealing with COVID-19 patients. However, only 30.95% thought about making an excuse or taking a holiday to avoid working during the pandemic.

**Table 2:** Participants responded to COVID-19 pandemic related questions.

COVID-19 pandemic related questions	Answer	Number (n)	Percentage (%)
**Have you been diagnosed with COVID-19?**	Yes	6	3.57
	No	162	96.43
**Have your family or friend been diagnosed with COVID-19?**	Yes	57	33.93
	No	111	66.07
**Did you get sufficient infection prevention training for COVID-19?**	Yes	86	51.20
	No	82	48.80
**Do you think the current protection can prevent you from being infected?**	Yes	82	48.80
	No	86	51.20
**Are you worried about insufficient preventive measures to protect against COVID-19 virus?**	Yes	128	76.19
	No	40	23.81
**Are you worried about being infected?**	Yes	113	67.26
	No	55	32.74
**At the place you work in, is there an extra precaution are taken in the radiology departments?**	Yes	134	79.76
	No	34	20.24
**Are you worried about the current prevention and control strategy?**	Yes	120	71.43
	No	48	28.57
**As a radiological staff, do you think that working in the radiology field have increased your level of cautions regarding facing COVID-19 patients?**	Yes	153	91.07
	No	15	8.93
**Do you think that the people around you started to worry being with you once they know you work in a radiological field?**	Yes	124	73.81
	No	44	26.19
**Have you tried or thought about getting an excuse or holiday and avoid working during this pandemic?**	Yes	52	30.95
	No	116	69.05

The results of the GAD-7 indicate that radiology staff were affected by different degrees of anxiety during the pandemic. Of the 168-radiology staff that participated in the study, about 46.43% experienced no symptoms of anxiety, while 27.38%, 15.48%, 10.71% experienced mild, moderate, and severe symptoms of anxiety, respectively (Table 3).

**Table 3:** Severity level of anxiety, depression, and insomnia among radiology staff.

Psychological symptoms	Level of severity	Number (n)	Percentage (%)
**Anxiety**	Minimal/no	78	46.43
	Mild	46	27.38
	Moderate	26	15.48
	Sever	18	10.17
**Depression**	Minimal/no	72	42.86
	Mild	50	29.76
	Moderate	28	16.67
	severe	18	10.71
**Insomnia**	None	89	52.98
	subthreshold	54	32.14
	Moderate	21	12.50
	Severe	4	2.38

Sleep difficulty was measured by the ISI. This study demonstrated that 32.14%, 12.50%, and 2.38% of the radiology staff had sub-threshold, moderately severe, and severe symptoms of insomnia, respectively (Table 3). The prevalence of depression among radiology staff was measured by PHQ-9. The results demonstrate that 29.76%, 16.67%, and 10.71% of radiology staff had mild, moderate, and severe symptoms of depression, respectively.

Participants reported that they did not receive any psychological support materials, such as brochures, leaflets, or books from their hospital during the pandemic (83.33%) or any online psychological assistance via social media and other platforms (77.98%). The final question was meant to assess their current health status compared to their health before the pandemic. 58.33% believed there was no change, while 30.95% thought that their health had worsened (Table 4).

**Table 4:** Available resources of mental healthcare services during pandemic.

Psychological services received during COVID-19 pandemic	Answer	Number (n)	Percentage (%)
**Psychological materials such as brochures, leaflets, books provided by psychological staff and gave it to you in the hospital**	Yes	28	16.67
	No	140	83.33
**Psychological resources available in media such psychologist gave on online psychological assistance techniques through social media or TV**	Yes	37	22.02
	No	131	77.98
**Individual psychotherapy or group therapy**	Yes	24	14.29
	No	144	85.71
**How do you assess your current health status compared to your health before the pandemic?**	Getting better	10	5.95
	Almost unchanged	98	58.33
	Worse	51	30.95
	Much worse	8	4.76

The results showed that the mean (Standard Deviation) for insomnia, depression, and anxiety symptoms among the participants were 8.5 (5.82), 6.67 (5.55) and 6.66 (5.15), respectively. When comparing the three symptoms among individuals with varying genders, ages, and levels of work experience, the results only demonstrated a significant difference in depression with working experience and age (Table 5). Post-hoc analysis comparison showed that depression was lower in individuals older than 35 years old compared to those between those between 20 to 25 years old with a mean difference of 4.6 (1.60–7.61). The same pattern was found for depression among those individuals with different levels of work experience. Those with more experience (>35) had significantly lower levels of depression than those with less experience (<5). The mean difference was 3.84 (1.13–6.55) (Table 6).

**Table 5:** Statistical analysis for mental health disorders based on gender, age, and working experience.

	Variable	Categories	Mean	Std. Deviation	P value
**Gender**	**Insomnia**	Male	8.46	5.77	0.92
		Female	8.55	5.91	
	**Anxiety**	Male	6.04	4.77	0.1
		Female	7.33	5.49	
	**Depression**	Male	5.93	5.46	0.67
		Female	7.49	5.57	
**Age**	**Insomnia**	20–25	8.32	5.66	0.37
		26–35	8.97	5.96	
		>35	7.24	5.58	
	**Anxiety**	20–25	7.55	5.87	0.36
		26–35	6.40	4.69	
		>35	6.07	5.37	
	**Depression**	20–25	8.64	6.33	0.002*
		26–35	6.51	5.23	
		>35	4.03	4.02	
**Working experience**	**Insomnia**	<5	8.63	5.99	0.6
		5–10	8.89	5.61	
		>10	7.50	5.49	
	**Anxiety**	>5	6.89	5.22	0.64
		5–10	6.59	4.96	
		>10	5.86	5.24	
	**Depression**	<5	7.66	5.94	0.003*
		5–10	5.97	4.66	
		>10	3.82	3.87	

* Statistically significant P-value < 0.05.

**Table 6:** Post hoc analysis of age and working experiences variables.

	Variable	Comparison	Mean Difference in depression symptoms	Sig.	95% Confidence Interval	
					Lower Bound	Upper Bound
**Age**	**20–25**	26–35	2.13	0.07	-0.14	4.39
		>35	4.6	0.00	1.60	7.61
	**26–35**	>35	2.48	0.08	-0.22	5.17
**Working experience**	**<5**	5–10	1.69	0.23	-0.74	4.12
		>10	3.84	0.00	1.13	6.55
	**5–10**	>10	2.15	0.25	-1.05	5.35

## Discussion

This study demonstrated that 91.07% of the participants believed that working in the radiology field had increased their level of caution with regard to dealing with COVID-19 patients. During the pandemic, 53.57% of the participants suffered from anxiety, 47.02% had symptoms of insomnia, and 57.14% had mild to severe depression.

Interestingly, this study found that only 30% of the participants tried to or considered making excuses to avoid work during the pandemic. This shows the altruism and moral heroism of the radiology staff and other healthcare professionals as well as the great sacrifices they made by decreasing attention on themselves and focusing on the patients. Tsamakis et al. reported that healthcare professionals during the COVID-19 pandemic have been working under immense psychological pressures and have high rates of psychiatric morbidity, similar to the conditions witnessed during the H1N1 and SARS outbreaks [28]. More than half of healthcare professionals during the H1N1 outbreaks experienced moderately high anxiety [29]. During SARS, healthcare professionals reported cognitive symptoms of anxiety [30]. A recent study in China demonstrated that 34.8% of medical staff suffered from anxiety during the COVID-19 pandemic [31], which is consistent with our study. Radiology staff have experienced anxiety due to the ongoing COVID-19 outbreak. The results of this study may be explained by the fact that the increasing number of suspected and infected COVID-19 patients, as well as the increasing number of countries affected by the pandemic, have made radiology staff increasingly concerned about becoming infected (67.26%), which, therefore, increased their anxiety levels. Moreover, because of the high demand for healthcare, radiology staff have experienced longer work shifts, often with limited safety precautions in place. Even though radiology departments have taken extra precautions to protect staff, more than 70% of the radiology staff are worried about the current prevention and control strategies to protect themselves against COVID-19. Balkhi et al. reported that high levels of anxiety could lead to suspicion and dissatisfaction with the preventive measures taken by the government [32].

The increased work hours of healthcare professionals also produced significantly poorer sleep quality, which has resulted in insomnia [18]. During the SARS outbreak, the prevalence of insomnia was 37% among healthcare professionals [33]. The same situation is repeating during this pandemic, as Zhang et al. found that more than one-third of healthcare professionals had insomnia symptoms [18]. Furthermore, Azzez et al. and Abdulah & Musa found that 45.5% and 68.3% of physicians had difficulty maintaining regular sleep patterns [34, 35]. Consistent with the literature, the current study found that 47.02% of radiology staff in Saudi Arabia also experience symptoms of insomnia ranging from mild to severe. Radiology staff should wear full-body Personal Protective Equipment (PPE) when dealing with confirmed cases of COVID-19, including double face masks, isolation caps, double-layer gloves, foot covers, and goggles that may cause physical discomfort and difficulty breathing [17]. In these risky circumstances, radiology staff become physically and mentally exhausted, which diminishes their sleep quality due to excessive stress. Sleep and the circadian system play an important role in regulating immune functionality [36]. It is important for radiology staff to get enough sleep during this pandemic, as a good night’s sleep is one of the greatest means of defending against the virus and enhancing one’s immunity [37].

It has been reported that healthcare workers who suffer from insomnia probably feel depressed [18]. Depression is a result of an extreme or consistent stress that has not been handled, mostly because people struggle to manage stressful life events [38, 39]. Nowadays, the COVID-19 pandemic is a source of extreme stress for the population of the entire world [18]. A Chinese study found that 50.7% of healthcare professionals had depressive symptoms [40]. This study also found that 57.05% of radiology staff feel depressed, which demonstrated a positive relationship between mental health disorders (depression, anxiety, or a combination of both) and frequent exposure to social media throughout the COVID-19 pandemic [41]. Thus, social media could be a possible explanation for the increase in depression levels among radiology staff. Social media is not always a reliable source of information about the pandemic [41]. It often provides false information, rumors, and fake news reports that result in a misinformation overload [42], which can, in turn, impact mental health. Another possible explanation for this trend might be that the increasing mortality rate among healthcare workers is causing more radiology staff to feel depressed.

The results of this study demonstrated that radiology staff who are older than 35 years old had lower levels of depression. This result is consistent with previous studies that found a negative correlation between age and levels of depression [20]. Younger adults demonstrated high levels of mental health disorders as they have fewer adaptive skills in response to stressors and biopsychosocial changes related to age could play a role in increasing stress levels [43, 44]. Another important finding was that mental health disorders have a negative relationship on work experience. Participants with more the 35 years of working experience had less symptoms of depression. This result is consistent with the previous study, which showed that junior nurses experienced high levels of mental health symptoms during the pandemic [45]. A review study also demonstrated that healthcare providers with more professional experience were less likely to develop psychiatric symptoms [46]. A possible explanation is that senior workers might have experienced a previous pandemic and, as a result, have the ability to more effectively deal with emergency situations than their younger counterparts.

Several factors mentioned previously could impact the mental health of radiology staff, in terms of anxiety, insomnia, and depression. The fear of possibly transmitting the virus to their colleagues, friends, and families, sudden changes in lifestyle and social distancing, uncertain guidelines for case management, and other ambiguities in this pandemic have undoubtedly led to anxiety, insomnia, and depression [32, 47].

The present study has demonstrated the limited availability of mental healthcare services, including brochures, leaflets, books, and media-based psychological resources, provided to radiology staff by psychiatric departments in hospitals. During the Ebola crisis, the lack of psychosocial and mental health assistance systems as well as appropriate trained mental health professionals, increased the risk of psychological distress [48]. Today, it is necessary to implement effective mental health supports as soon as possible to alleviate the impact of COVID-19 and improve psychological resilience. Interventions that can fight the COVID-19 pandemic and minimize the risk of mental health disorders include: reasonable rest, less shift work, standard safety protocols in radiology departments, training in specialized protection for those dealing with COVID-19 patients, and comprehensive direction on the use of PPE [19]. Other measures that can be taken to maintain mental health include avoiding extreme exposure to social media and news and maintaining a healthy diet and lifestyle, which can boost immunity and keep one’s mood elevated [49]. Secure services that offer psychological counselling through virtual clinics, chatlines, hotlines, as well as online psychoeducation and therapy sessions are also recommended [14]. Regular screening for anxiety, insomnia, and depression should be performed for radiology staff. It is important to the maintain mental and physical health for radiology staff during the COVID-19 pandemic [19].

## Conclusion

The findings of this study demonstrate that a large percentage of radiology staff in Saudi Arabia have been suffering from mental health disorders, such as anxiety, insomnia, and depression during the COVID-19 pandemic. There is a limited availability of mental healthcare services for radiology staff, such as brochures, leaflets, and/or psychological resources, available in media. Immediate intervention to establish mental health support systems should be provided to radiology staff to alleviate their stress and enhance their overall mental health. It is important to prepare for future pandemics caused by infectious diseases by developing mental healthcare systems to care and protect radiology staff who may suddenly find themselves on the highly risky front line in the battle against the disease. Moreover, future research could explore the mental health of radiology staff working in other countries and implement the same measurements to explore outcomes.

## Limitations

Even though this is the first study to focus on the mental health of radiology staff during the COVID-19 pandemic, the study has several limitations. First, it includes self-designed questions in the sections related to the perception risk of COVID-19 since no standard questionnaire has explored risk perception during the pandemic. This could lead to misreporting of data and some biased responses. Second, because it was an online survey, it was difficult to contact non-social media users, which affectively generalize the results. Third, the study is cross-sectional and does not examine the efficiency of mental health services. Because it only measured at specific period and the possibility of changes in post-trauma mental health, a longitudinal study is required. Finally, the histories of mental health disorders among the radiology staff were not considered, which may lead to confounding biases.

## Supporting information

S1 File Demographic characteristics of participants. (XLSX)

S2 File Participants responded to COVID-19 pandemic. (XLSX)

S3 File Severity level of anxiety, depression, and insomnia. (XLSX)

S4 File Analysis on gender, age, and working experience. (XLSX)
